# Ramp lesions: a systematic review of MRI diagnostic accuracy and treatment efficacy

**DOI:** 10.1186/s40634-020-00287-x

**Published:** 2020-09-25

**Authors:** José Moreira, Margarida Almeida, Nuno Lunet, Manuel Gutierres

**Affiliations:** 1 Faculty of Medicine, University of Porto, Alameda Hernâni Monteiro, 4200-319 Porto, Portugal; 2 EPIUnit—Instituto de Saúde Pública, University of Porto, Porto, Portugal; 3 Departamento de Ciências da Saúde Pública e Forenses e Educação Médica, Faculty of Medicine, University of Porto, Porto, Portugal; 4 Serviço de Ortopedia e Traumatologia, Centro Hospitalar de S. João, Porto, Portugal

**Keywords:** Ramp lesions, Anterior cruciate ligament, Knee instability, Magnetic resonance imaging, Meniscal repair

## Abstract

**Purpose:**

We conducted a systematic review of the published literature to assess the accuracy of Magnetic Resonance Imaging (MRI) in establishing the presence of ramp lesions (RLs) in Anterior Cruciate Ligament (ACL) deficient knees and the clinical efficacy of the surgical repair of RLs.

**Methods:**

A comprehensive search of the MEDLINE, Web of Science and Scopus databases was performed according to PRISMA guidelines. Studies assessing MRI diagnostic accuracy for RLs or the clinical effect of RL repair in participants with ACL injuries were included. Diagnostic accuracy measures were pooled and plotted in forest plots. Preoperative and at last follow-up treatment efficacy outcome measures were extracted and plotted in forest plots, for graphical comprehension.

**Results:**

Sixteen studies met the criteria and were included. The diagnostic analysis showed a pooled sensitivity, specificity, positive and negative likelihood ratios of 65.1% (95% CI, 59.73 to 70.42), 91.6% (95% CI, 89.14 to 94.05), 2.91 (95% CI, 2.38–3.55) and 0.53 (95% CI, 0.44–0.64), respectively, with high heterogeneity (I^2^ above 80%) for all measures. Treatment analysis showed improved Lysholm Knee Score, IKDC score and laxity difference between the knees in all studies after meniscal suture repair. A separate analysis showed no differences between repair of smaller, stable, RLs with meniscal sutures and repair with abrasion and trephination only.

**Conclusion:**

Although the results present considerable heterogeneity, MRI seems to demonstrate moderate accuracy in the diagnosis of RLs in patients with ACL tear and the surgical repair of RLs can be associated with improved overall outcomes.

## Introduction

Primarily described in 1983 by Hamberg et al. [30], injury to the peripheral attachment of the posterior horn of the medial meniscus (PHMM) after Anterior Cruciate Ligament (ACL) lesion (termed “Ramp Lesion”, by Strobel et al. [77]) still remains an understudied topic.

The coexistence of ACL rupture and other knee injuries has been described in many studies. Acute ACL rupture is associated with a meniscal injury in over 50% (16–82%, in different studies) of injuries and over 80% of chronic ACL ruptures [10, 29, 35, 49, 62, 82, 83]. The medial meniscus is firmly attached to the tibia and femur, allowing it to act as a knee stabilizer, preventing excessive anterior translation, especially in the ACL-deficient knee, thus being especially susceptible to injuries after ACL lesion [1, 6, 13, 17, 42, 70, 78].

Ramp Lesions (RL) are also often described as meniscocapsular separations and meniscosynovial tears [13]. Recent literature has extended the definition to include injuries of the meniscotibial ligament and peripheral longitudinal tears in the Red-Red zone of the PHMM [12, 13, 60, 72, 79, 80] and different classification systems have been proposed by Thaunat et al. [79] and Seil et al. [64].

The epidemiology of RLs is still incompletely defined. Incidence ranges from 9% to 40% in many small population studies, becoming higher with chronicity of ACL deficiency [1, 8, 11, 67, 71]. Male sex, younger age, chronic (> 6 weeks) ACL injury, increased time from injury, presence of a lateral meniscal tear and increased medial meniscal slope are significant risk factors for RLs [44, 65, 73].

When a RL is present in an ACL-deficient knee, anterior and external rotational laxities are significantly increased, compared to isolated ACL injury. In such cases, repair of the ACL alone does not fully correct this abnormality, suggesting the importance of diagnosing and repairing the meniscal injury during ACL reconstruction [51, 70, 76]. However, clinical identification can be a troublesome situation. There are no specific clinical tests for the diagnosis of RLs and common tests for meniscal tears are not accurate in diagnosing this injury [55].

While MRI can be a reliable diagnostic modality for most meniscal pathologies [31], its sensitivity and specificity for the diagnosis of RL have been questioned by some authors, marking the need for further research, especially for a quantitative analysis of data from the existing studies [11, 38, 55].

The general consensus is that arthroscopic evaluation is necessary to reliably assess the occurrence of a RL after ACL injury [13]. Standard anterolateral arthroscopy portals, even with the addition of probing, have limited accuracy, requiring insertion of the arthroscope in the posteromedial recess, using the Intercondylar (or Gillquist) view or a posteromedial portal [24, 54, 63, 72].

What to do when a RL is identified is not consensual and may depend on whether ACL injury is acute or chronic. Once they are located in a vascularized region of the meniscus, several authors have stated that shorter or more stable tears may be managed with conservative treatment following ACL reconstruction, especially in the acute setting [57, 68]. Conversely, some authors state that acute repair is necessary since the hypermobility of the detached meniscocapsular structure delays, or even impedes, spontaneous healing [1, 4, 13, 75].

Repair options may include open repair, termed posteromedial arthrotomy [21, 30], now widely replaced by other techniques, using posteromedial approaches (with a hook) or an anteromedial approach (all-inside or inside out repair techniques) [15, 60, 77]. For small and stable subacute or chronic injuries, stimulation of a healing response with abrasion and trephination may be recommended [60]. The existing literature lacks a comprehensive analysis of data from the existing treatment studies, in order to clearly understand the benefit of repairing the meniscal tear.

The purpose of this systematic review was to assess the published literature with regard to the diagnosis and treatment of RLs in ACL deficient knees in order to describe the accuracy of MRI (compared to arthroscopy) in establishing the presence of a RL and the clinical efficacy of the surgical repair of RLs, by evaluating the difference between preoperative and postoperative knee scores. Our hypothesis was that MRI cannot adequately diagnose RLs and surgical repair of ramp lesion leads to improved clinical outcomes at final follow-up.

## Materials and methods

The present study was conducted according to the Preferred Reporting Items for Systematic Reviews and Meta-Analyses (PRISMA) guidelines [50]. A protocol for the conduction of the review was written before the start of the study and followed until the end of the review.

### Study eligibility

*Types of studies*: all study designs, except for case reports, ex vivo studies, reviews and technical notes, were included, without publication date, status or language restrictions.

*Participants*: studies were considered when they examined participants, of any age, with acute or chronic ACL rupture undergoing (or who underwent) reconstruction and at risk for or diagnosed with a concomitant RL.

*Interventions and Comparisons*: studies were included if they compared the diagnostic accuracy of MRI with arthroscopy (gold standard) or if they assessed the clinical effect of RL repair (through any method of repair).

*Outcomes*: primary outcomes considered were sensitivity, specificity and likelihood ratios (LR) for the diagnostic studies and Lysholm Knee Score, International Knee Documentation Committee (IKDC) Score and laxity difference between the affected and the non-affected knees, for the treatment analysis. Articles not presenting any of the aforementioned outcomes or without a pre-treatment analysis of patients were excluded.

### Literature search

Included databases were MEDLINE, Web of Science and Scopus. The last search was run on 12/01/2020 and search clauses can be found in appendix. The search terms cover a broad spectrum of meniscus and associated knee injuries, to avoid missing relevant literature. As a result of the different designations of RLs, keywords such as “ramp”, “hidden”, “meniscocapsular”, “meniscosynovial” and “posteromedial” were included in the search clause. The use of additional limiters and filters was restricted, in order to avoid missing potentially relevant studies. The reference lists of the selected articles were also checked for relevancy.

### Study selection and data abstraction

Two researchers independently screened the titles and abstracts yielded by the database searches against the inclusion criteria. Disagreements were solved by consensus. Full reports for all titles and abstracts that appeared to have met the inclusion criteria or where there was some uncertainty were sought. Full text reports were then screened and included if they met the inclusion criteria. Reasons for excluding papers were recorded. None of the researchers was blinded to the journal titles, authors or institutions.

Data regarding the study sample and methodology, intervention details (MRI and surgical techniques), and all reported important outcomes were systematically extracted from the included studies, following the predefined protocol.

### Risk of Bias assessment

The Quality Assessment of Diagnostic Accuracy Studies (QUADAS-2) instrument was used to assess possible risk of bias in diagnostic studies, according to the Cochrane Collaboration recommendation [61]. Each of the 11 recommended quality items was judged as ‘yes’ or ‘no’, according to whether that characteristic was present. When there was insufficient detail reported in the study, that item was judged ‘unclear’.

Quality of the included articles of the treatment studies was assessed using the Methodological Index for Non-Randomized Studies (MINORS) [69], a validated instrument, designed for non-randomized surgical studies, and based on 12 items, the last four specific for comparative studies. Each item was scored as “0”, “1” or “2”, if the item was not reported, reported but inadequate or reported and adequate, respectively.

Quality assessment was accomplished by one of the authors. Results are presented for each item, independently.

### Data analysis

Sensitivity, specificity and positive and negative LRs, with corresponding 95% confidence intervals (CI), were extracted whenever provided in the original reports, or computed with the available information. The diagnostic accuracy measures were pooled and analysed using a random effects model and plotted in forest plots. Statistical heterogeneity was quantified using the I^2^ statistic.

Preoperative and at last follow-up treatment efficacy outcome measures were extracted and plotted in forest plots for graphical comprehension of the results. No meta-analysis of these results was attempted, since no measures of association were provided in most studies. A separate analysis with two studies [45, 85] was performed to compare the efficacy of meniscal suture to abrasion and trephination only, in small (< 1.5 cm) and stable RLs.

Unless otherwise noted, continuous variables were expressed as means and 95% CI and categorical variables were expressed as frequencies. Standard deviations were used to estimate 95% CI, when CI were not provided.

Stata software (version 15.1) was used for the meta-analysis and to produce forest plots. A *P* < 0.05 was considered statistically significant.

## Results

### Literature search

**Fig. 1:**
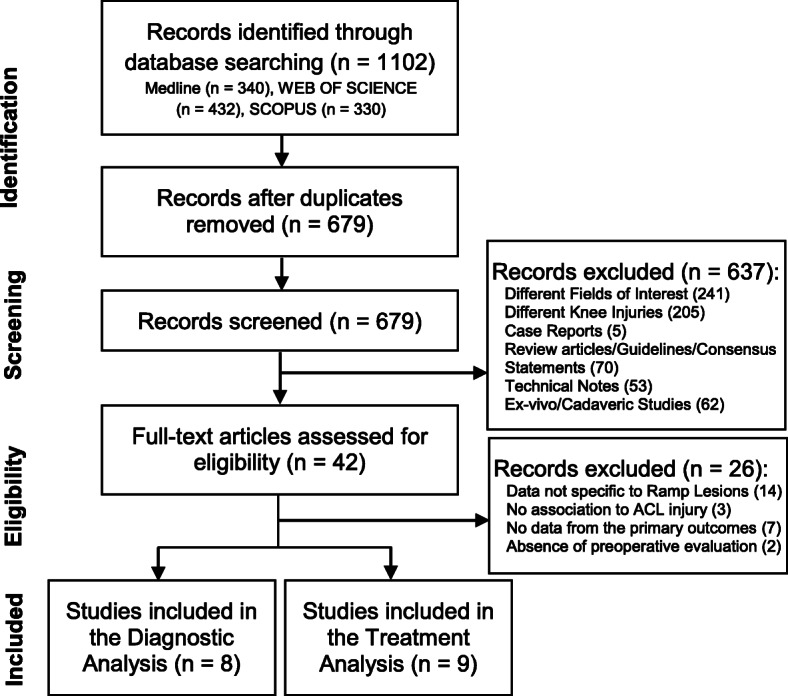
Study selection process for the Systematic Review using the PRISMA (Preferred Reporting Items for Systematic Reviews and Meta-Analyses) guidelines. Gulenc et al. [28] was included in both portions of the analysis

The systematic review flow chart is presented as Fig. 1. Initial search through the databases retrieved 1102 articles. A total of 16 original research articles were included in the systematic review, eight studies were included in the diagnostic analysis and nine studies were included in the treatment analysis, with one study [28] being included in both portions.

### Characteristics of the included studies

The study and patient characteristics from the included studies are summarized in Table 1. All studies were conducted in a single centre and evaluated a total of 1959 patients. Populations depicted in the studies presented a predominance of males (except in one study by Furumatsu et al. [26]) and young adults.

**Table 1:** Study and Patient Characteristics^a^

Author (Year)	Study Period	Design		***N***	Age, y^b^	Male, %	Focus
Arner (2017) [7]	2013 to 2015	P/NC		90	28 ± 10 (14–45)	50.0	D
Chen (2018) [14]	Aug/2010 to Dec/2014	R/C		46	26 (18–41)	73.9	T
DePhillipo (2017) [22]	April/2010 to July/2016	P/C		301	29.6 ± 12.5 (14–61)	66.0	D
Furumatsu (2014) [26]	July/2009 to Dec/2011	P/C		20	19 (15–38)	40.0	T
Gulenc (2019) [28]	2017	P/NC		15	26.8 (18–35)	53.3	D/T
Hatayama (2018) [32]	April/2013 to Aug/2017	P/C		155	25.3 (13–60)	51.0	D
Keyhani (2017) [36]	2011 to 2014	P/C		128	24 (18–48)	83.6	T
Kim (2018) [38]	June/2011 to April/2015	P/C		195	31.7 ± 11.7	88.2	D
Kumar (2018) [40]	Jan/2006 to June/2016	R/C		178	NR.	NR.	D
Li (2015) [43]	Aug/2011 to Feb/2014	P/C		23	NR.	NR.	T
Liu (2017) [45]^c^	Aug/2008 to April/2012	P/C	(SG)	50	35.6 ± 8.5	76	T
			(AG)	41	34.8 ± 9.1	73.2	
Malatray (2018) [48]	Oct/2014 to May/2016	P/C		56	14.0 ± 1.3 (12–17)	76.8	D
Sonnery-Cottet (2018) [74]	Jan/2013 and Aug/2015	R/C		383	27.4 ± 9.2 (14–60)	76.5	T
Thaunat (2016) [80]	Oct/2012 to March/2013	P/C		132	26.4 (12–57)	83.3	T
Yang (2017) [85]^c^	Jan/2010 to Jan/2014	R/C	(SG)	37	35.7 ± 8.5	75.7	T
			(AG)	31	34.8 ± 8.1	74.2	
Yeo (2018) [86]	Jan/2015 to Sep/2017	R/C		78	37.3 (19–52)	82.1	D

^a^*AG* abrasion and trephination group, *Aug* August, *D* diagnosis, *Dec* December, *Feb* February, *Jan* January, *NR* not reported, *Oct* October, *P* prospective, *R* retrospective, *Sep* September, *SG* meniscal suture group, *T* treatment, *Y* years

^b^Age is expressed as mean ± SD (Range), when available

^c^Liu et al. [45] and Yang et al. [85] used 2 different cohorts to compare different treatment approaches

The MRI characteristics of the diagnostic studies included are summarized in Table 2. Hatayama et al. [41] used two cohorts in their study to compare different magnet strengths in the diagnosis of RL (3.0-Tesla versus 1.5-Tesla). MRI diagnostic criteria are similar in all but one study by Kumar et al. [40], where they used oedema of the tibial plateau as a marker of RLs. Sagittal fat-suppressed proton density-weighted image and fat-suppressed T2-weighted image were the preferred sequences. Only two studies [22, 32] reported MRI interpretation simultaneously by a musculoskeletal radiologist and an orthopaedic surgeon, the remaining reported MRI interpretation by either a radiologist or a surgeon only. The estimated time from injury to the diagnostic MRI was not mentioned in any of the studies.

**Table 2:** MRI characteristics of the Studies included in this review^a^

Author (Year)	Knee Position	Magnet Strength, T	Slice Thickness & MRI Sequence	RLs, %	Diagnostic Criteria
Arner (2017) [7]	Near full extension.	1.5	3 mm; Sequences NR.	14.4	High SI or separation between the posterior capsule and the PHMM.
DePhillipo (2017) [22]	NR.	3.0 or 1.5	NR; Sag. PDFS and T2FS.	16.6	High SI or separation between the posterior capsule and the PHMM.
Gulenc (2019) [28]	NR.	NR.	NR; Sagittal T2FS.	NR.	Separation between the capsule and the PHMM or tibial oedema.
Hatayama (2018) [32]^b^	Near full extension.	3.0 (*N*= 59)	2 mm; Sag. PDFS.	20.3	High SI or separation between the posterior capsule and the PHMM.
		1.5 (*N*= 96)	NR.	37.8	
Kim (2018) [38]	NR.	NR.	NR; Sag. PDFS.	25.6	Peripheral LT ≤ 4 mm of the meniscocapsular junction of the PHMM.
Kumar (2018) [40]	NR.	NR.	NR; Sag. PDFS and T2FS.	14.9	Oedema of the posterior medial tibial plateau.
Malatray (2018) [48]	Near full extension.	NR.	NR.	23.2	Peripheral LT of the meniscocapsular junction of the PHMM.
Yeo (2018) [86]	Neutral	3.0 or 1.5	3–4 mm; Sag. PDFS and T2FS.	9.0	High SI or separation between the posterior capsule and the PHMM.

^a^*LT* longitudinal tear, *MRI* Magnetic Resonance Imaging, *NR* not reported, *PDFS* Fat-suppressed Proton Density-weighted image, *PHMM* posterior horn of the Medial Meniscus, *RLs* proportion of ramp lesions, *Sag* Sagittal, *SI* fluid-like Signal Intensity, *TFI* time from injury, *T2FS* fat-suppressed T2-weighted image, *T* Tesla

^b^Hatayama et al. [32] used 2 cohorts to compare different magnet strengths in the diagnosis of ramp lesions

Table 3 compiles the treatment approaches from the studies included in the review. ACL reconstruction was performed in all patients, either by a hamstring autograft (640 patients), a patellar bone-tendon-patellar bone autograft (98 patients) and a quadriceps tendon graft (two patients). The ACL reconstruction strategy was absent in three studies [28, 36, 43]. Sonnery-Cottet et al. [74] added anterolateral ligament repair to the intervention in 189 patients. All studies present different postoperative rehabilitation protocols, with many common key points. All patients were followed for a minimum of 12 months, except in the study by Gulenc et al. [28] (33 weeks).

**Table 3:** Treatment Methods from the Studies included in this review^a^

Author (Year)	Surgery Details & ACL Graft	Postoperative Protocol	TFI to Repair	Follow-up Time	Adverse Events
Chen (2018) [14]	All-inside suture device (*FasT-Fix*). HT.	0°-90° at 4 wks; full WB/ROM in 6 wk.; full activity at 6 mo.	NR.	32 mo.	2 MFC cartilage injuries.
Furumatsu (2014) [26]	All-inside suture device (*FasT-Fix*). BPTB, HT.	Partial WB in 2 wk.; full WB in 4–6 wk.; full activity in 5–8 mo.	6 mo.	24 mo.	5% secondary interventions.
Gulenc (2019) [28]	All-inside suture technique. NR.	0–90° by the 3rd wk.; full activity in 4–6 mo.	NR.	33.1 ± 12.7 wk.	NR.
Keyhani (2017) [36]	All-inside suture with hook. NR.	0°-90° and partial WB after 2–4 wk.; full WB and ROM at 6 wk.	NR.	>24 mo.	Residual joint pain in 3 pts.
Li (2015) [43]	All-inside suture device (*FasT-Fix*). NR.	0°-90° by the 4th wk.; full WB in 6 wk.; full activity after 6 mo.	NR.	14 mo.	NR.
Liu (2017) [45]	All-inside suture with hook. HT.	0°-90° by the 4th wk.; full WB at 4 wk.; full activity at 9–12 mo.	NR.	37.9 ± 15.9 mo.	NR.
Sonnery-Cottet (2018) [74]	All-inside suture with hook. BPTB, HT.	0°-90° by the 4th wk.; WB as tolerated; full activity at 8–9 mo.	13.5 ± 32 mo.	37.4 ± 9 mo.	NR.
Thaunat (2016) [80]	All-inside suture with hook. HT, BPTB, QT.	0°-90° by the 6th wk.; full WB in 3 wk.; full activity at 9 mo.	NR	27 mo.	2 hematomas needing lavage.
Yang (2017) [85]	All-inside suture device (*FasT-Fix*). HT.	Partial WB at 8 wks; full WB at 12 wk.; full activity after 6 mo.	45.2 ± 28.1 d	>24 mo.	Residual joint pain in 3 pts.
Liu (2017) [45]	Abrasion and trephination. HT.	0°-90° by the 4th wk.; full WB at 4 wk.; full activity at 9–12 mo.	NR.	40.3 ± 16.5 mo.	NR.
Yang (2017) [85]	Abrasion and trephination. HT.	Partial WB at 8 wks; full WB at 12 wk.; full activity after 6 mo.	42.8 ± 25.4 d	>24 mo.	Residual joint pain in 2 pts.

^a^*ACL* Anterior Cruciate Ligament, *BTB* bone-tendon-bone autograft, *d* days, *MFC* medial femoral condyle, *mo* months, *HT* hamstring tendon autograft, *NR* not reported, *pts* patients, *QT* quadriceps tendon autograft, *ROM* range of motion, *TFI* time from injury, *WB* weight-bearing, *wk* weeks

### Risk of Bias assessment

**Fig. 2:**
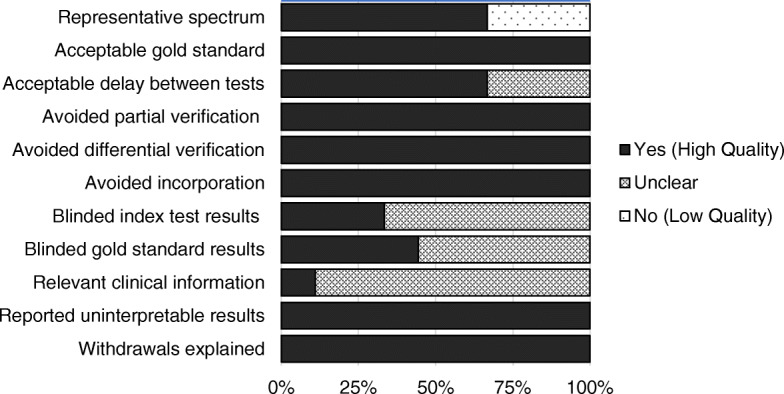
Risk of bias of the diagnostic studies, using the QUADAS-2 tool

Regarding risk of bias in the diagnostic studies, portrayed in Fig. 2, all studies satisfied at least six of the 11 items recommended by the QUADAS-2 tool. Three studies were considered of low quality concerning the representativeness of the spectrum of patients, as a result of the study of a paediatric population [48] or the study of patients already diagnosed with Ramp Lesions [22, 28]. The interval between MRI and the reference standard was absent in three studies [38, 40, 48]. Blinding of the two tests results was only reported in three studies [7, 22, 40] and only one study [86] reported on the clinical information available at the time of interpretation of test results. All other topics were considered of high quality for every study.

Table 4 summarizes risk of bias in the treatment studies according to the MINORS tool. Liu et al. [45] was not included in the quality assessment, as it was designed as a randomized controlled trial. This study was assessed to have a low overall risk of bias, according to the randomization process, blinding of the allocated intervention and unbiased outcome measurements. Blinding of the interventions to the investigators assessing the outcomes was only performed in one study, by Sonnery-Cottet et al. [74], as was the case for prospective calculation of the study sample, performed only in the study by Keyhani et al. [36].

**Table 4:** Risk of bias for treatment studies using the MINORS tool

Studies	Item 1	Item 2	Item 3	Item 4	Item 5	Item 6	Item 7	Item 8	Item 9	Item 10	Item11	Item 12
Chen et al. [14]	1	2	0	2	0	2	2	0	–	–	–	–
Furumatsu et al. [26]	2	2	1	2	0	2	2	0	–	–	–	–
Gulenc et al. [28]	1	1	0	2	0	1	2	0	–	–	–	–
Keyhani et al. [36]	2	2	0	2	0	2	2	2	–	–	–	–
Li et al. [43]	0	2	0	1	0	2	2	0	–	–	–	–
Sonnery-Cottet et al. [74]	2	2	2	2	2	2	1	0	2	2	1	2
Thaunat et al. [80]	2	2	0	2	0	2	2	0	–	–	–	–
Yang et al. [85]	2	2	1	2	0	2	2	0	2	2	2	2

**Item 1 - Aim:** clearly stated aim; **Item 2** - **Consecutiveness of Patients:** all patients fit for inclusion have been included; **Item 3** - **Prospective Collection:** data collected according to a pre-established protocol; **Item 4** - **Appropriate Endpoints:** endpoints appropriate to the aim of the study; **Item 5** - **Endpoint Assessment:** unbiased blinded assessment; **Item 6** - **Follow-up Period:** appropriate to the aim of the study**; Item 7 - Losses to follow up:** less than 5%; **Item 8** - **Study Size Calculation:** prospective calculation of the study size. Additional criteria for comparative studies: **Item 9** - **Control Group:** adequate control group; **Item 10** - **Contemporary groups:** both groups managed in the same time period; **Item 11** - **Baseline Equivalence**: similar groups; **Item 12** - **Statistical Analyses:** in accordance with the type of study. 0: not reported; 1: reported but inadequate; 2: reported and adequate

### Diagnostic accuracy of MRI

**Fig. 3:**
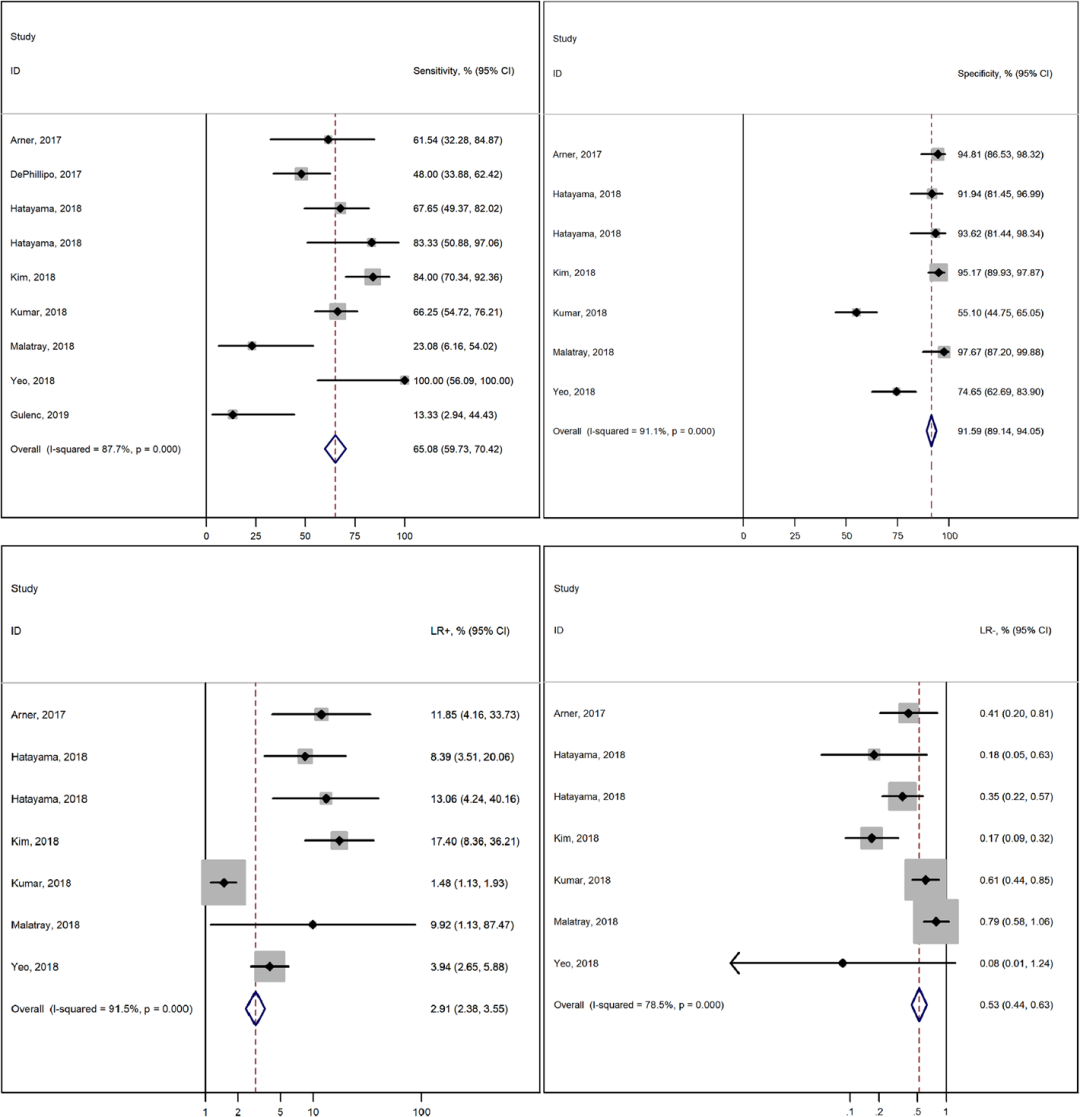
Forest plots summarizing MRI accuracy in the detection of ramp lesions. Dots in squares represent the estimated measures while the horizontal lines represent the 95% CI. The diamond shape represents the combined estimate. I^2^ with 95% CI and the result of the using the chi-squared test are also provided. Hatayama et al. [32] used 2 different cohorts to compare different magnet strengths, 3-Tesla (upper) and 1,5-Tesla (lower)

Figure 3 depicts the forest plots summarizing the accuracy of MRI in the detection of RLs. The pooled results showed a sensitivity of 65.08% (95% CI, 59.73 to 70.42), a specificity of 91.59% (95% CI, 89.14 to 94.05), a positive LR of 2.91 (95% CI, 2.38 to 3.55) and a negative LR of 0.53 (95% CI, 0.44 to 0.64). Heterogeneity was high, with I^2^ statistics above 80% for all outcomes evaluated.

### Treatment efficacy of ramp lesion repair

**Fig. 4:**
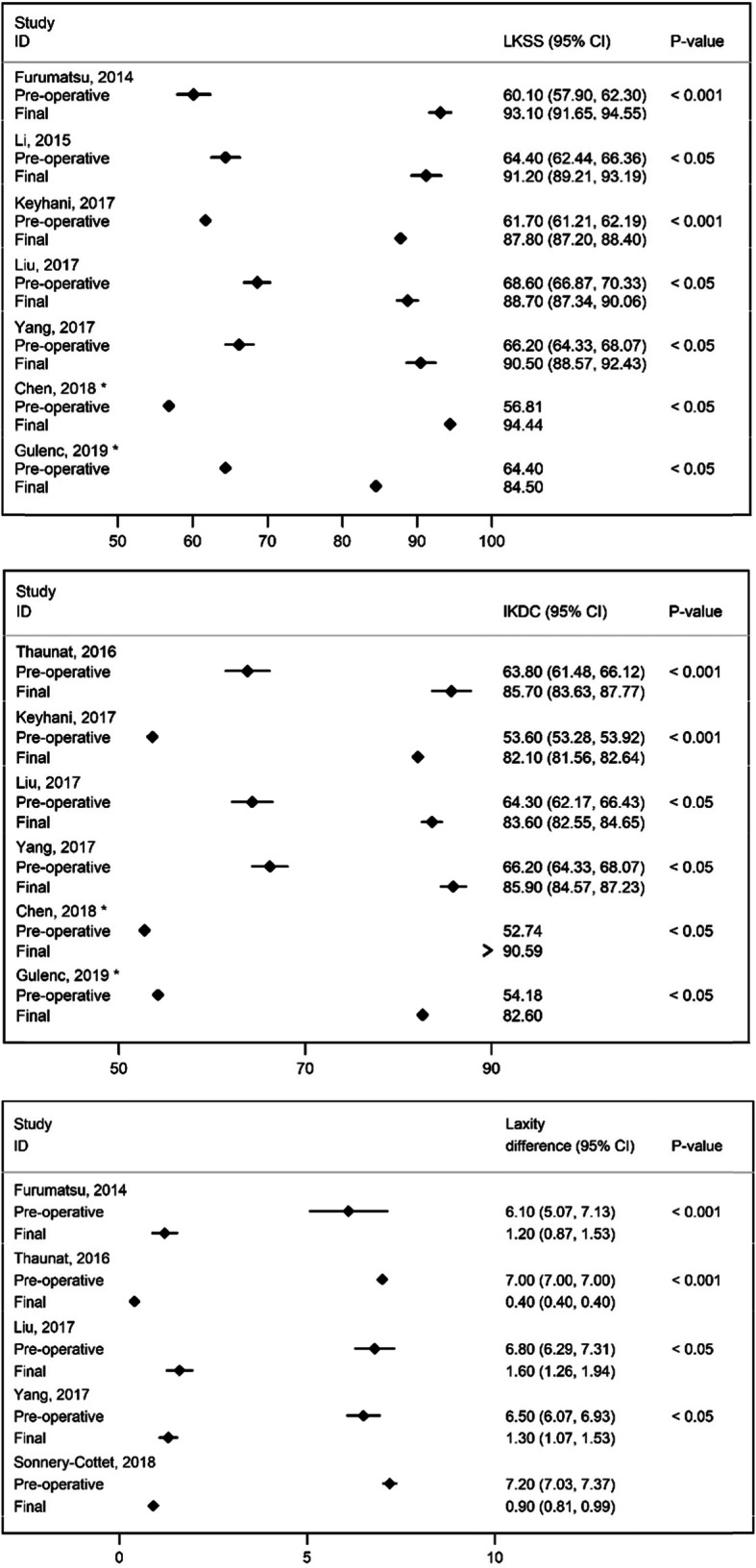
Forest plots grouping the mean Pre-operative and Final (at final follow-up) Lysholm Knee Scores, International Knee Documentation Committee scores and laxity differences between the affected and the unaffected knee. Dots in squares represent the estimated measures while the horizontal lines represent the 95% CI. *only point estimates are presented because no confidence intervals or information to compute them were available from these studies

Figure 4 shows the forest plots describing the results from studies that evaluated the effects of treatment. Mean preoperative and final Lysholm Knee Scores ranged from 56.8 to 68.6 and 84.5 to 94.4, respectively. Mean preoperative and final IKDC scores ranged from 52.7 to 64.3 and 82.1 to 90.6, respectively. Mean preoperative and final laxity differences between the affected and the unaffected knees ranged from 6.1 mm to 7.2 mm and 0.4 mm to 1.6 mm, respectively. The improved final outcomes are statistically significant in all studies (*P* < 0.05), using tests for paired samples.

**Fig. 5:**
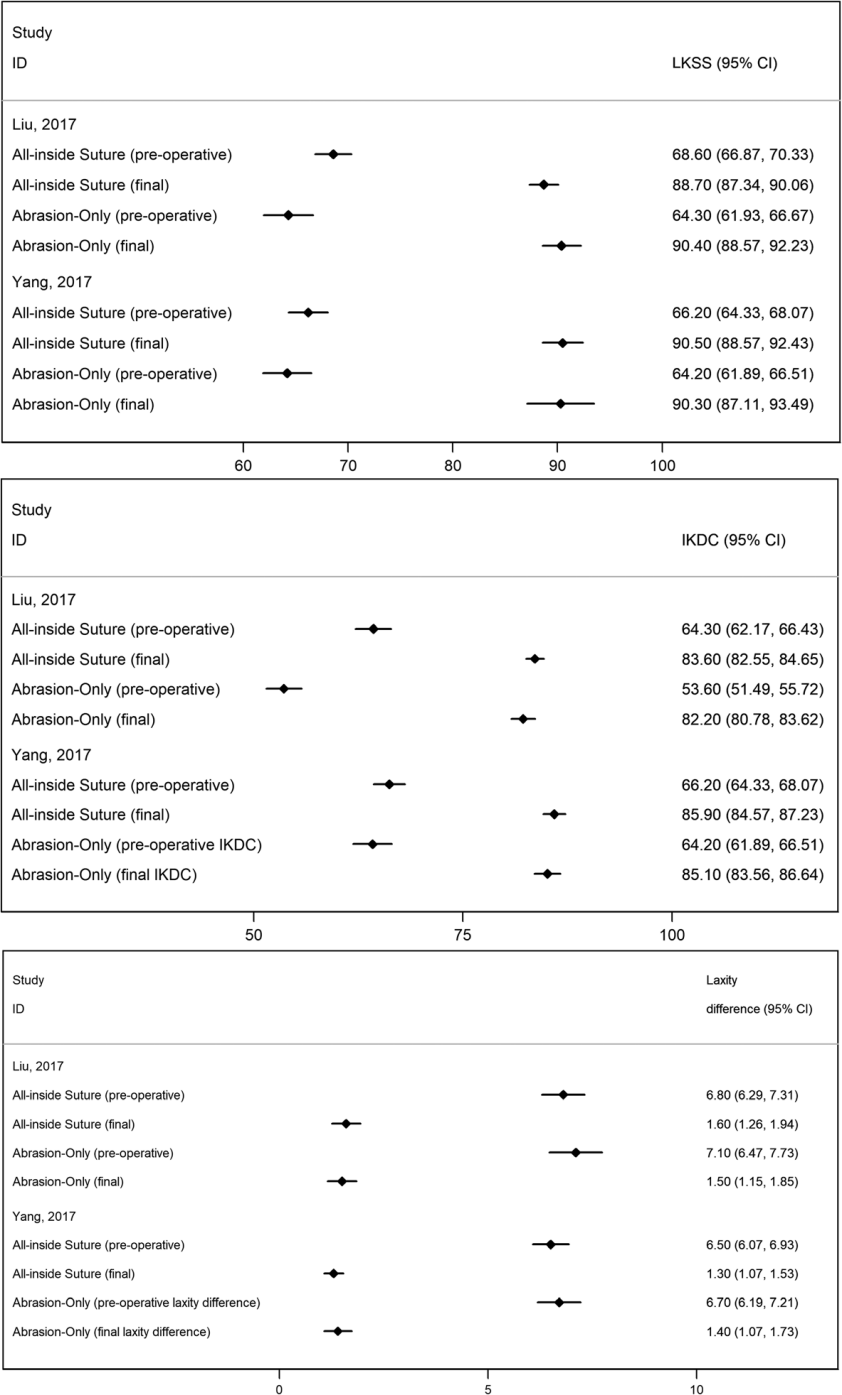
Forest plots comparing the mean Preoperative and Final (at final follow-up) outcomes between all-inside suture of the medial meniscus versus abrasion and trephination for the repair of small and stable Ramp Lesions (< 1.5 cm), in the two studies that evaluated both techniques. Dots in squares represent the estimated measures while the horizontal lines represent the 95% CI

Figure 5 presents the comparison of the all-inside suture technique of the medial meniscus versus abrasion and trephination for the repair of small and stable Ramp Lesions (< 1.5 cm), in the two studies that evaluated both techniques. Lysholm Knee Scores, IKDC scores and laxity differences between the affected and the unaffected knees in both groups increased significantly postoperatively (*P* < 0.05), but no significant differences were observed between the two groups before or after the surgery (*P* > 0.05) in both studies.

## Discussion

The results of this review demonstrated that MRI has a moderate sensitivity (65%) and a high specificity (92%) in the diagnosis of RL. The positive and negative LR (2.91 and 0.53, respectively) indicate a questionable clinical significance of the MRI, as the pre-test probability will only suffer slight (around 15%) modifications after MRI interpretation.

MRI has been appointed as a reliable diagnostic modality for most medial meniscal pathologies, with sensitivities of over 90% and specificities of over 80%, in two systematic reviews with meta-analysis [18, 31, 53]. This accuracy for the diagnosis of medial meniscus injury is said to be lower in the presence of an ACL tear [20, 52], which may explain the lower sensitivity of MRI for the diagnosis of RLs found in this review. DePhillipo et al. [23] inquired 36 directors of orthopaedic sports medicine through an electronic questionnaire and found that despite 89% of surgeons stated that they routinely use MRI for the diagnosis, 50% believed that they are rarely or only sometimes accurate in the diagnosis [23]. In fact, our results suggest that MRI may have a good accuracy in the diagnosis of RLs, but arthroscopy remains the reference standard and should not be replaced by MRI, as stated in the literature for other cartilage damages in the knee [18, 25, 53].

Our results showed that Lysholm Knee Scores, IKDC scores and laxity difference between the affected and the unaffected knees significantly improve after RL repair with sutures. The two studies which compared all-inside suture and abrasion and trephination of the meniscus in small and stable RLs (< 1.5 cm) found no significant differences between the two methods, suggesting that in these cases, abrasion and trephination may be a viable option for the management of RLs [45, 85].

Medial meniscus repair associated with ACL repair has been associated with lower rates of meniscectomy and osteoarthritis [16, 46, 66]. Moreover, repair of injuries to the PHMM in the context of ACL reconstruction has been associated with high success rates, when evaluated by second-look arthroscopy (complete healing ranging from 82.1 to 96.4%), with little complications and satisfactory clinical results [3, 4]. Results from this review showed that the surgical repair leads to improved clinical results compared to preoperative scores, congruent with the results from medial meniscal repair of other injuries. Despite the absence of a meta-analysis of these results, because no effect measures for direct comparisons between the pre and post treatment periods were provided in the original reports, the visual presentation of the results in forest plots provides a good picture of the benefit of surgery and differs from previous reviews [5, 12].

It is generally accepted that extensive medial meniscal injuries require surgical repair (with inside-out or all-inside sutures) and 92% of surgeons reported to surgically repair meniscal RLs in their clinical practice [23]. On the other hand, there is some controversy in the management of small (< 1.5–2 cm) and stable (with little anterior translation of the PHMM from the anteromedial portal) meniscal tears [58, 68, 84]. In the two studies included in this review, comparing all-inside sutures to abrasion and trephination of the meniscus, the overall outcomes were similar, but larger population studies are warranted.

### Limitations

The present systematic review has a few limitations that should be discussed. This review analyses data of a relatively small number of studies. Regardless of the comprehensiveness of the search expressions, the use of multiple databases and the inclusion of articles in several languages, the available literature on this topic is scarce and some of the articles failed to report important outcomes (such as, sensitivity and specificity for diagnostic studies [8, 11, 37, 39, 72] and preoperative plus postoperative clinical outcomes [2, 34, 41, 73] for treatment studies) and had to be excluded.

Both the diagnostic and treatment studies included are heterogeneous regarding the methods used. Different magnet strengths, different knee position, differences in the diagnostic criteria and differences in the arthroscopic protocol could be responsible for the differences in sensitivity and specificity and for the heterogeneity encountered, in the diagnostic studies. The evidence regarding the accuracy of different magnet strengths in the diagnosis of meniscal injuries is conflicting [27, 47, 59, 81], and although 3-Tesla MRI appears to be superior to 1.5-Tesla, a recent meta-analysis showed no statistically significant difference between the two resolutions in sensitivity and specificity [56]. There are no defined criteria to diagnose RLs on MRI, but irregular posterior meniscal outline and fluid separating the meniscus and capsule, are considered to correlate best with the diagnosis of RLs [19, 31] and may explain the conflicting results found by Kumar et al. [40]. Considering patient position, Bollen [11] hypothesized that when the knee is in near full extension, meniscocapsular separation is reduced, making the diagnosis harder and affecting the sensitivity of MRIs. Finally, all studies reported arthroscopy as the gold-standard for the diagnosis of RLs, but the arthroscopic protocol was heterogenous. Two studies reported [22, 40] assessment of RLs by probing from anterolateral portals, while two studies [28, 32] reported adding a posteromedial portal to the protocol if an injury was suspected during probing from the anterolateral portal and three studies [7, 38, 48] reported using posteromedial portals in every patient (Yeo et al. [86] did not specify the portals used during arthroscopy). Although, the evidence regarding the accuracy of different portals in the diagnosis of RLs during arthroscopy is conflicting [24, 38, 44, 72], the adoption of different arthroscopic protocols by the studies may have affected our findings.

In the treatment studies, differences between the surgery and postoperative protocols could also be responsible for some variability in the results. To our knowledge, no study has compared the efficacy of all-inside suture using a device (anteromedial approach) with all-inside suture using a hook (posteromedial approach). Visual inspection of the forest plot conveys the impression that outcomes between the two methods are similar, but a more objective approach, with direct comparison of the two methods, is important and missing in the literature. Sonnery-Cottet et al. [74] performed anterolateral ligament reconstruction in 189 patients, but no significant differences were found between the two groups, regarding clinical scores. Also, there was variability between the postoperative rehabilitation protocols adopted by each article and, even though most share the same basic principles, as prevention of excessive weightbearing and joint compressive forces (that lead to disruption of meniscal healing [9, 33, 55, 75]), a standardization of the postoperative protocol is needed for future research.

The studies included in this review were also heterogeneous regarding the amount of information provided. Mean time from injury to the diagnostic MRI was absent in all the diagnostic studies and time from MRI to arthroscopy was missing in three [38, 40, 48]. RLs may heal spontaneously, causing a mismatch between the MRI and arthroscopic findings if there is substantial delay between the two methods. The amount of clinical information available to the radiologist at the time of MRI interpretation was omitted in most articles. As combination of clinical and MRI findings provides the most accurate non-invasive method currently available for diagnosing injuries of the menisci [18], this information is crucial and should be reported in future studies. Time from injury to surgery (and distinction between acute or chronic injuries) was also absent in many treatment studies. As chronicity of the lesion can be a factor in the decision of treatment, this information must also be provided in future studies.

The studies that addressed the effects of treatment present many quality issues. Most studies [14, 28, 36, 43, 80] were uncontrolled before-after studies with a single preoperative outcome measurement, presenting a serious risk of bias, as the observed improvements cannot be reliably attributed to the intervention, instead of other factors, as the natural history of meniscal healing. The absence of blinding was also common across the reviewed studies and contributes to an increased risk of bias.

Thus, further radiological studies are warranted using standardized optimal conditions (as knee positioning and MRI sequences evaluated) and the inclusion of clinical findings in the evaluation of the images, possibly leading to the development of preoperative diagnostic algorithms. Also, further studies comparing different surgical options and the non-surgical management of these injuries are warranted to make assertions regarding the correct approach in the management of these conditions.

## Conclusion

Notwithstanding the longevity of recognition of RLs, risk factors for developing this type of injury, the incidence, diagnosis and the outcomes of treatment remain incompletely defined. Although the results present considerable heterogeneity and the quality could be improved, MRI seems to demonstrate moderate accuracy in the diagnosis of RLs in patients presenting with acute or chronic ACL tear and the surgical repair of can be associated with improved overall outcomes. A continued interest in the development of knowledge of this condition is essential.

## Appendix

### Database query string for PubMed

(tibial meniscus injuries[Mesh] AND (“ramp” OR “hidden” OR “meniscocapsular” OR “meniscosynovial” OR “posteromedial” OR (“medial” AND “peripheral”))) OR ((“Anterior Cruciate Ligament Injuries”[Mesh] OR “meniscus”[Tiab] OR “meniscal”[Tiab]) AND (“ramp”[Tiab] OR “hidden”[Tiab]) AND “lesion”[Tiab]) OR (“meniscocapsular”[Tiab] OR “meniscosynovial”[Tiab] OR ((“meniscus”[Tiab] OR “meniscal”[Tiab]) AND ((“peripheral”[Tiab] AND “medial”[Tiab]) OR “posteromedial”[Tiab])) AND (lesion[Tiab] OR “tear”[Tiab] OR “separation”[Tiab])).

### Database query string for Scopus and Web of Science

TITLE-ABS-KEY ((ramp AND lesion) OR (hidden AND lesion) AND (meniscus OR meniscal OR (Anterior AND Cruciate AND Ligament))) OR (meniscocapsular OR meniscosynovial OR ((meniscus OR meniscal) AND posteromedial) AND (separation OR tear OR lesion OR injury)).

## Abbreviations

ACL: Anterior Cruciate Ligament
CI: Confidence Interval
IKDC: International Knee Documentation System
LR: Likelihood Ratio
MINORS: Methodological Index for Non-randomized Studies
MRI: Magnetic Resonance Imaging
PHMM: Posterior horn of the Medial Meniscus
PRISMA: Preferred Reporting Items for Systematic Reviews and Meta-Analyses
QUADAS-2: Quality Assessment of Diagnostic Accuracy Studies
RL: Ramp lesion
